# Cabibbo–Kobayashi–Maskawa-favored *B* decays to a scalar meson and a *D* meson

**DOI:** 10.1140/epjc/s10052-017-5441-1

**Published:** 2017-12-14

**Authors:** Zhi-Tian Zou, Ying Li, Xin Liu

**Affiliations:** 1 Department of Physics, Yantai University, Yantai, 264005 China; 2 School of Physics and Electronic Engineering, Jiangsu Normal University, Xuzhou, 221116 China

## Abstract

In this work, we attempt to study the Cabibbo–Kobayashi–Maskawa-favored $B \rightarrow \overline{D} S$ (“*S*” denoting the scalar meson) decays within the perturbative QCD approach at the leading order and the leading power. Although the light scalar mesons are widely perceived as primarily the four-quark bound states, in practice it is hard for us to make quantitative predictions based on the four-quark picture for light scalars. Hence, we calculate the decays with light scalars in the two-quark model. For the decays with scalar mesons above 1 GeV, we have explored two possible scenarios, depending on whether the light scalars are treated as the lowest lying $q\bar{q}$ states or four-quark particles. In total, we calculated the branching fractions of 72 decay modes, and most of them are in the range $10^{-4}$–$10^{-7}$, which are measurable in the on-going LHCb experiment and the forthcoming Belle-II experiment. Moreover, since in the standard model these decays occur only through tree operators and have no *CP* asymmetries, any deviation will be a signal of new physics beyond the standard model. Despite large uncertainties induced by nonperturbative parameters and corrections of high order and high power, our results and discussions will be useful for the on-going LHCb and the forthcoming Belle-II experiments.

## Introduction

Even though the quark–antiquark model works well for the pseudoscalar mesons and vector mesons, the study of the inner substructure of the scalar mesons is quite non-trivial, because the conventional quark–antiquark model cannot explain the properties, such as the decay rates and the mass spectrum, especially for ones below 1 GeV. Therefore, the understanding of the internal structure of the scalar mesons is one of the most interesting topics in hadron physics.

The scalar mesons reported by experiments include the isosinglet $f_0(600)$($\sigma$), $f_0(980)$, $f_0(1370)$, $f_0(1500)$ and $f_0(1710)$, the isodoublet $K_{0}^{*}(800)$($\kappa$) and $K_{0}^{*}(1430)$, and the isovector $a_0(980)$ and $a_0(1450)$ [1]. Studies of the mass spectrum of scalar mesons and their strong as well as electromagnetic decays suggest that the scalar mesons with the mass below 1 GeV constitute one nonet, while those near 1.5 GeV form another one [2–6]. Irrespective of the existence of the $\sigma$ and the $\kappa$ mesons, in the literature, the scalar mesons have been identified as ordinary $\bar{q}q$ states, four-quark states or meson–meson bound states or even those supplemented with a scalar glueball. Unfortunately, we have not reached a definite conclusion yet till now, due to the unknown nonperturbative properties of QCD, though many efforts have been made to interpret the quark contents of the scalar mesons [7–11]. Now, a consistent picture [2] provided by the data suggests that the scalar meson states above 1 GeV can be identified as a conventional $q\bar{q}$ nonet with some possible glue content, which has widely been accepted. However, the quark structure of the light scalar mesons below or near 1 GeV has been quite controversial, though they are widely perceived as primarily four-quark bound states. For example, $f_0(980)$ has been treated as a traditional $q\overline{q}$ state [12–14], as a four-quark $qq\overline{q}\overline{q}$ state [15, 16], and even as a bound state of hadrons [17, 18]. In fact, even in the two-quark picture, the quark component is still unclear; for example, the observation of $D_s \rightarrow f_0(980) \pi ^{+}$ decay introduced the probability of the $s\overline{s}$ component of $f_0(980)$, while $\Gamma (J/\psi \rightarrow f_0(980)\omega )\sim \Gamma (J/\psi \rightarrow f_0(980)\phi )$ indicated the existence of the non-strange components [19, 20]. Therefore, if the light scalar mesons are dominated by two-quark states, the isoscalars $f_0(980)$ and $f_0(600)$ perhaps should be mixing states like the $\eta$–$\eta ^{\prime }$ system [21, 22]. In the literature, according to the category that the light mesons belong to, there are two typical scenarios for describing the scalar mesons [5]. The scenario 1 (S1) is the naive two-quark model: the nonet mesons below 1 GeV, such as $\kappa$, $a_0(980)$, $f_0(980)$, and $\sigma$, are treated as the lowest lying states, and those near 1.5 GeV, such as $a_0(1450)$, $K_0(1430)$, $f_0(1370/1500)$, are the first orbitally excited states. In scenario 2 (S2), the nonet mesons near 1.5 GeV are viewed as the lowest lying states, while the mesons below 1 GeV may be the exotic states beyond the quark model such as four-quark bound states.

In hadron physics, most studies of the light scalar mesons are concentrated on the decay properties of the scalar mesons and the production of the scalar mesons in $p\overline{p}$ (or *np*) collisions or the $\phi$ radiative decays [23]. After the first *B* decay into a scalar meson, $B\rightarrow f_0(980)K$, was observed by Belle [24] and confirmed by BaBar [25], the studies of the scalar mesons in hadronic *B* decays have attracted more attention because of the large phase space of the *B* decays. Theoretically, from the studies of QCD sum rules, one finds that the nonperturbative parameters, such as decay constants and the distribution amplitudes, are related to the certain scenarios seriously, which affect the experimental observables noticeably, such as branching fractions and CP asymmetries. So, by comparing the experimental data and the theoretical predictions, one could deduce which scenario is favorable. Motivated by this viewpoint, many charmless *B* decays with scalars have been studied extensively [5, 6, 26–47].

Recently, the LHCb collaboration reported their first measurements of the decays $B_{(s)} \rightarrow \overline{D} f_0(980)$ and $\overline{D}\sigma$ [48, 49],

1 $$\begin{aligned}&Br(B^0 \rightarrow \overline{D}^0 \sigma )\nonumber \\&\quad =(11.2\pm 0.8\pm 0.5\pm 2.1\pm 0.5)\times 10^{-5},\nonumber \\&Br(B^0 \rightarrow \overline{D}^0 f_0(980))\nonumber \\&\quad =(1.34\pm 0.25\pm 0.10\pm 0.46\pm 0.06)\times 10^{-5},\nonumber \\&Br(B^0_s \rightarrow \overline{D}^0 f_0(980))\nonumber \\&\quad =(1.7\pm 1.0\pm 0.5\pm 0.1)\times 10^{-6}. \end{aligned}$$

Note that in these three decay modes, not only the scalars but also the charmed mesons are involved, and these decays are induced by the $\bar{b}\rightarrow \bar{c}$ transition. Very recently, in Ref. [50], we have attempted to study the $B\rightarrow D^{(*)}S$ decays induced by the $\overline{b} \rightarrow \overline{u}$ transition within the perturbative QCD (PQCD) approach, which are suppressed by the Cabibbo–Kobayashi–Maskawa (CKM) matrix element $|V_{ub}|$, but which evade the suppression by the vector decay constants of the scalar mesons. Now, since some experimental data are available, it is worthwhile for us to extend our study to the CKM favored $B \rightarrow \overline{D}^{(*)} S$ decays induced by the $\overline{b}\rightarrow \overline{c}$ transition.

To handle the hadronic effects for $B \rightarrow \overline{D}^{(*)} S$ decays, we use the factorization formalism, called perturbative QCD approach [51–54], which is based on the $k_T$ factorization and the transition matrix element is described by the convolution of hadron wave functions and the hard kernel. In the limit of heavy quarks, in order to guarantee that color transparency mechanism is satisfied, i.e., no soft gluon exchange occurring between the final states, we argue that the following hierarchy must be postulated [55, 56]:

2 $$\begin{aligned} m_B \gg m_{D^{(*)}} \gg \overline{\Lambda }\end{aligned}$$

with $$\overline{\Lambda }=m_B-m_b\sim m_{D^{(*)}} -m_c\sim \Lambda _\mathrm{QCD}$$. The relation $$m_B \gg m_{D^{(*)}} $$ justifies the perturbative analysis of the $$ B \rightarrow \overline{D}^{(*)} $$ form factors at large recoil and the definition of light-cone $$ \overline{D}^{(*)}$$ meson wave functions. The relation $$m_{D^{(*)}} \gg \overline{\Lambda }$$ justifies the power expansion in the parameter $$\overline{\Lambda }/m_{D^{(*)}}$$. The small ratio $$\overline{\Lambda }/m_B$$ is regarded as being of higher power. Because of the inclusion of parton transverse degrees of freedom, large double logarithmic corrections $$\alpha _s \ln ^2 k_T$$ appear and should be summed to all orders. It turns out that the resultant Sudakov factor for an energetic $$D^{(*)} $$ meson is similar to that for a *B* meson. Including the Sudakov effects from $$k_T$$ resummation and from threshold resummation for hard amplitudes, the end-point singularities do not exist, and soft contributions can be suppressed effectively. Although the applicability PQCD is still in controversy [57–60], it has been employed for studying the two-body charmed *B* decays, such as $$B \rightarrow DP, DV, DA, DT$$ decays [56, 61–66, 97], where *P*, *V*, *A*, *T* denote the pseudoscalar, vector, axial-vector, and tensor mesons, respectively. Most of the predictions were in good agreement with the present experimental data. Compared with $$B\rightarrow D^{(*)}S$$ decays, for some $$B \rightarrow \overline{D}^{(*)}S$$ decays, the factorizable amplitude will vanish or will be heavily suppressed due to the vanishing or tiny vector decay constants of the scalar mesons. However, the hard-scattering emission diagrams and annihilation type diagrams perhaps provide sizable contributions; such cases are similar to the $$B\rightarrow \overline{D}^{(*)}P, V, T$$ decays [56, 61–66, 97]. We thus expect that the branching fractions of some decays are large enough to be measured in the current LHC experiment and/or the forthcoming Belle-II in the future. It is worth pointing out that the annihilation type diagrams can be perturbatively calculated in the PQCD approach without end-point singularity, and the predictions of some pure annihilation decays are well in agreement with data, such as the $$B_s \rightarrow \pi ^{+}\pi ^{-}$$ and $$B^0\rightarrow D_s^- K^+$$ [67–69].

It should be stressed that although many experimental data indicate that the light scalar mesons, such as $f_0(980)$ and $a_0(980)$, are predominately four-quark states, in practice it is very difficult for us to make quantitative predictions on $B\rightarrow DS$ based on the four-quark picture for *S*, because both the decay constants and the distribution amplitudes of *S* are beyond the conventional quark model. Hence, in practice we shall assume the two-quark scenario for light scalar mesons in the current work.

This paper is organized as follows: we will give a brief review of the formalism of the PQCD approach and specify the wave functions of the initial and final states in Sect. 2. The perturbative calculations and the analytic formulas are given in Sect. 3. The numerical results and phenomenological discussions will be presented in Sect. 4. The final section is reserved for the summary.

## Formalism and wave function

As aforementioned, based on the $k_T$ factorization, PQCD approach can effectively avoid the end-point singularity by keeping the intrinsic transverse momenta of inner quarks. The kept transverse momenta will introduce the additional energy scale and lead to the double logarithms appearing in the QCD radiative corrections, which can be resummed into the Sudakov factor. As a result, the Sudakov factor will suppress the soft region contribution and make the calculation in the PQCD approach reliable and consistent.

The effective Hamiltonian $H_\mathrm{eff}$ related to the $B\rightarrow \overline{D}^{(*)} S$ decays is given as [70]

3 $$\begin{aligned} H_\mathrm{eff}=\frac{G_F}{\sqrt{2}}V^*_{cb}V_{ud(s)}[C_1(\mu )O_1(\mu )+C_2(\mu )O_2(\mu )], \end{aligned}$$

with the four-quark tree-diagram operators

4 $$\begin{aligned} O_1= & {} (\overline{b}_{\alpha } c_{\beta })_{V-A} (\overline{u}_{\beta } d(s)_{\alpha })_{V-A},\nonumber \\ O_2= & {} (\overline{b}_{\alpha }c_{\alpha })_{V-A} (\overline{u}_{\beta }d(s)_{\beta })_{V-A}, \end{aligned}$$

where $\alpha$ and $\beta$ are the color indices, and $(\overline{b}_{\alpha } c_{\beta })_{V-A}=\overline{b}_{\alpha }\gamma ^{\mu }(1-\gamma ^{5}) c_{\beta }$. $V_{cb}$ and $V_{ud(s)}$ are the CKM matrix elements. $C_{1,2}$ are the so-called Wilson coefficients at the renormalization scale $\mu$.

To deal with the hadronic *B* decays with multiple scales, the factorization hypothesis is usually adopted. The physics regime higher than the scale of the *W* boson mass $(m_W)$ is electroweak and can be calculated perturbatively. Using renormalization group techniques, we can evaluate the dynamical effects and obtain the Wilson coefficients from the $m_W$ scale to the *b* quark mass ($m_b$) scale. The physics between $m_b$ scale and the factorization scale (*t*) can be calculated perturbatively, which is the so-called hard kernel in the PQCD approach. The dynamics below the factorizable scale is soft and nonperturbative but universal, which can be described by the hadronic wave function. So, in the PQCD approach, the decay amplitude can be written as the convolution of the Wilson coefficients *C*(*t*), the hard kernel $H(x_i,b_i,t)$, and the hadronic wave functions [71, 72],

5 $$\begin{aligned} \mathcal {A}\sim & {} \int \mathrm{d}x_1 \mathrm{d}x_2 \mathrm{d}x_3 b_1 \mathrm{d}b_1 b_2 \mathrm{d}b_2 b_3 \mathrm{d}b_3 \nonumber \\&\times \, \mathrm {Tr}[C(t)\Phi _B(x_1,b_1)\nonumber \\&\times \,\Phi _{D }(x_2,b_2)\Phi _{S }(x_3,b_3) H(x_i,,b_i,t)S_t(x_i)e^{-S(t)},\nonumber \\ \end{aligned}$$

where $\mathrm {Tr}$ denotes the trace over Dirac and color indices, the $x_i(i=1,2,3)$ and $b_i$ are the longitudinal momentum fractions and conjugate variables of $k_{Ti}$ of the valence quarks in each meson, respectively. The threshold resummation of the double logarithms $\ln ^2x_i$ lead to the jet function $S_t(x_i)$ [73]. The aforementioned Sudakov factor $e^{-S(t)}$, coming from the resummation of the double logarithms, can suppress the soft dynamics effectively, i.e. the long distance contributions in the small $k_T$ region [74, 75].

In PQCD, the most important inputs are the wave functions. For the scalar meson, in the two-quark picture, the wave function can be defined as6with the lightlike vectors $n=(1,0,\mathbf 0 _T)$ and $v=(0,1,\mathbf 0 _T)$. $\phi _S$ and $\phi _S^{S,T}$ are the leading-twist and twist-3 light-cone distribution amplitudes respectively, where *x* is the momentum fraction of the “quark”. The leading-twist light-cone distribution amplitude $\phi _S(x,\mu )$ of the scalar meson has the general form [5, 6]

7 $$\begin{aligned} \phi _S(x,\mu )= & {} \frac{3}{2\sqrt{6}}x(1-x)\left[ f_S(\mu )\right. \nonumber \\&\left. +\,\overline{f}_S\sum _{m=1}^{\infty }B_m(\mu )C_m^{3/2}(2x-1)\right. ], \end{aligned}$$

with the Gegenbauer moments $B_m$ and the Gegenbauer polynomials $C_m^{3/2}$. For the twist-3 distribution amplitudes, we adopt the asymptotic forms for simplicity,

8 $$\begin{aligned} \phi _S^{S}=\frac{\overline{f}_S}{2\sqrt{6}},\quad \phi _S^T=\frac{\overline{f}_S}{2\sqrt{6}}(1-2x). \end{aligned}$$

The $f_S$ and $\overline{f}_S$ are the vector decay constant and scalar decay constant of the scalar meson, respectively. For the neutral scalar mesons ($\sigma$, $f_0$, and $a_0^0$), the vector decay constant vanishes due to the conservation of the vector current. However, the scalar decay constant $\overline{f}_S$, related by the equation

9 $$\begin{aligned} \overline{f}_S=\mu f_S,\quad \mu =\frac{m_S}{m_2(\mu )-m_1(\mu )}, \end{aligned}$$

remains finite. Note that the above parameters, $B_m$, $f_S$, and $\overline{f}_S$, depend on certain scenarios, for the numerical results of which one is referred to Refs. [5, 6].

For the neutral light scalars $\sigma$ and $f_0(980)$, in the two-quark model, much experimental evidence indicates that there is a mixing between $\sigma$ and $f_0(980)$, which is similar to the $\eta$–$\eta ^{\prime }$ system,

10 $$\begin{aligned} \left( \begin{array}{c} \sigma \\ f_0 \end{array}\right) =\left( \begin{array}{cc} \cos \theta &{}-\sin \theta \\ \sin \theta &{} \cos \theta \end{array}\right) \left( \begin{array}{c}f_n\\ f_s\end{array}\right) , \end{aligned}$$

with $f_n=(u\overline{u}+d\overline{d})/\sqrt{2}$ and $f_s=s\overline{s}$. For the mixing angle $\theta$, various experimental measurements have provided different values [76–78]. Recently, the LHCb has proposed the upper limit $|\theta |<30^{\circ }$ by the process $\overline{B}^0\rightarrow J/\psi f_0(980)$ [79]. Analyzing the present experimental implications, we prefer to adopt the two possible ranges of $25^{\circ }<\theta <40^{\circ }$ and $140^{\circ }<\theta <165^{\circ }$ [80–85]. It is noted that, for the $f_0(980)$ and $\sigma$ mesons, there are other interpretations, for example, the $\pi \pi$ generalized distribution amplitudes [86, 87].

For the $f_0(1370)$–$f_0(1500)$ system, according to Ref. [88] and neglecting the tiny contribution from the scalar glueball [89–91], the mixing form can be simplified as

11 $$\begin{aligned}&f_0(1370)=0.78f_n+0.51f_s,\nonumber \\&f_0(1500)=-0.54f_n+0.84f_s. \end{aligned}$$

For the initial *B* meson, neglecting the numerically suppressed Lorentz structure, the remaining leading order wave function can be decomposed as [92, 93]12with

13 $$\begin{aligned} \phi _B =\phi _B^+ ,\quad \overline{\phi }_B =(\phi _B^--\phi ^+)/\sqrt{2}. \end{aligned}$$

The contribution from $\overline{\phi }_B$ starts from the next-to-leading-power ${\bar{\Lambda }}/{m_B}$, which should be included together with other next-to-leading-power contributions in order to form a complete analysis. So, we here neglected it in the current work. The light-cone distribution amplitude $\phi _B(x,b)$ can be written as [92–94]

14 $$\begin{aligned} \phi _B(x,b)=N_Bx^2(1-x^2)\exp \left[ -\frac{m_B^2x^2}{2\omega }-\frac{1}{2}\omega _B^2b^2\right] , \end{aligned}$$

with the normalization constant $N_B$, which can be determined through the following normalization condition:

15 $$\begin{aligned} \int _0^1 \mathrm{d}x \phi _B(x,b=0)=\frac{f_B}{2\sqrt{6}}. \end{aligned}$$

For the shape parameter $\omega _B$ and the decay constant $f_B$, we will take $(0.4\pm 0.04)$ GeV and $(0.19\pm 0.02)$ GeV for the *B* meson, respectively, and take $(0.5\pm 0.05)$ GeV and $(0.23\pm 0.03)$ GeV for the $B_s$ meson, due to the SU(3) breaking effects [51–54].

In terms of the heavy quark limit, the two-parton light-cone distribution amplitudes of the $D(D^*)$ meson will be taken as [95–98]16
17with the distribution amplitudes [96–98]

18 $$\begin{aligned} \phi _D(x,b)= & {} \phi _{D^*}^{L,T}(x,b)=\frac{1}{2\sqrt{6}}f_{D^{(*)}}6x(1-x)[1\nonumber \\&+\,C_D(1-2x)]\exp \left[ -\frac{1}{2}\omega _D^2b^2\right] , \end{aligned}$$

with the shape parameter $\omega _D=0.15\pm 0.5$ GeV. Note that the high-twist distribution amplitudes are not included either, because they are suppressed by $\overline{\Lambda }/{m_{D^{(*)}}}$. We choose $C_D=0.5\pm 0.1$ and $f_D=207$ MeV for the *D* meson, and $C_D=0.4\pm 0.1$ and $f_{D_s}=241$ MeV for the $D_s$ meson [99]. The parameters $C_D$ are fitted from the $B\rightarrow DP(V)$ and $B_s\rightarrow D_sP(V)$ decays [96–98]. For $D_{(s)}^*$, the decay constants can be obtained through the relation based on the heavy quark effective theory, which can be found in Refs. [61–64, 97].

## Perturbative calculation

**Fig. 1:**
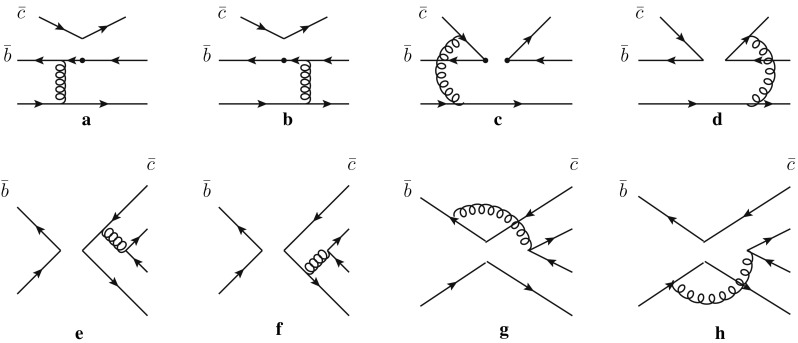
Leading order Feynman diagrams contributing to the $B\,\rightarrow \,\overline{D}^{(*)}S$ decays in PQCD approach

**Fig. 2:**
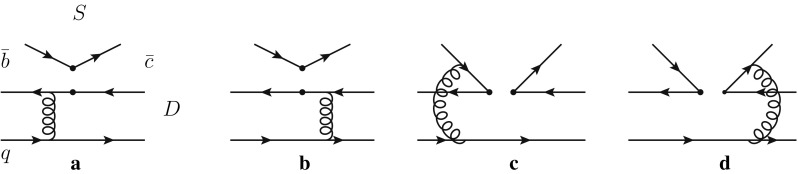
Leading order Feynman diagrams contributing to the $B\,\rightarrow \,\overline{D}^{(*)}S$ decays with a scalar meson emitted in PQCD

In this section, within the PQCD approach, we specifically calculate the decay amplitudes without the Wilson coefficients in Eq. (5) for each Feynman diagram, and we express the calculated amplitudes as a convolution of the hard kernel and the mesons’ wave functions. It is noted that there are two kinds of diagrams contributing to the considered decays at the leading order. The diagrams with a $\overline{D}$ meson emitted are presented in Fig. 1, and those with a scalar meson emitted are listed in Fig. 2.

For the two factorizable emission diagrams (a) and (b) in Fig. 1, the amplitudes can be written as

19 $$\begin{aligned} \mathcal {M}_\mathrm{ef}= & {} 8\pi C_f f_D m_B^4\int _0^1\mathrm{d}x_1\mathrm{d}x_3\int _0^{1/\Lambda }b_1\mathrm{d}b_1b_3\mathrm{d}b_3\phi _B(x_1,b_1)\nonumber \\&\times \, \left\{ \left[ \phi _S(x_3)(r_D^2(2x_3+1)-(x_3+1))\right. \right. \nonumber \\&\left. \left. +\,r_S(2x_3-1)(\phi _S^S(x_3)+\phi _S^T(x_3))\right] \right. \nonumber \\&\left. \cdot E_\mathrm{ef}(t_a)h_\mathrm{ef}(x_1,x_3(1-r_D^2),b_1,b_3)\right. \nonumber \\&\left. -\,2r_S\phi _S^S(x_3)E_\mathrm{ef}(t_b)h_\mathrm{ef}(x_3,x_1(1-r_D^2),b_3,b_1)\right\} , \end{aligned}$$

where $r_S=m_S/m_B$, $r_D=m_D/m_B$, and the color factor $C_f=4/3$ for *B* decays. The expressions for the scale *t*, the Sudakov factor *E*, and the hard functions *h* from the denominator of the propagators can be found in Appendix A of Ref. [63].

The two diagrams (c) and (d) in Fig. 1 are the so-called hard-scattering emission diagrams. Compared to the previous two, each decay amplitude involves three meson wave functions. After integrating out $b_3$ with the $\delta$ function $\delta (b_1-b_3)$, the amplitudes for these two diagrams can be expressed by

20 $$\begin{aligned} \mathcal {M}_\mathrm{enf}= & {} 16\sqrt{\frac{2}{3}}\pi C_f m_B^4\int _0^1\mathrm{d}x_1\mathrm{d}x_2\mathrm{d}x_3\nonumber \\&\times \int _0^{1/\Lambda }b_1\mathrm{d}b_1b_2\mathrm{d}b_2\phi _B(x_1,b_1)\phi _D(x_2,b_2)\nonumber \\&\times \Bigg \{[\phi _S(x_3)(r_D^2-r_S^2(2x_2+x_3-2)+x_2-1)\nonumber \\&+\,r_Sx_3(\phi _S^S(x_3)-\phi _S^T(x_3))]E_\mathrm{enf}(t_c)h_{enf1}(x_i,b_i)\nonumber \\&-\,[\phi _S(x_3)(x_2(r_D^2+2r_S^2-1)+x_3(2r_D^2+r_S^2-1))\nonumber \\&+\,r_Sx_3(\phi _S^S(x_3)+\phi _S^T(x_3))]E_\mathrm{enf}(t_d)h_\mathrm{enf2}(x_i,b_i)\Bigg \}. \nonumber \\ \end{aligned}$$

The four diagrams in the second row are the annihilation type diagrams, which can be perturbatively calculated in the PQCD approach. Diagrams (e) and (f) are the factorizable diagrams with the *B* meson factorized out, and the amplitudes can be written as

21 $$\begin{aligned} \mathcal {M}_{af}= & {} 8\pi C_f f_B m_B^4\int _0^1\mathrm{d}x_2\mathrm{d}x_3\nonumber \\&\times \int _0^{1/\Lambda }b_2\mathrm{d}b_2b_3\mathrm{d}b_3\phi _D(x_2,b_2)\nonumber \\&\times \Bigg \{[\phi _S(x_3)(r_D^2(2x_3-3)+(r_S^2-1)(x_3-1))\nonumber \\&+\,r_Dr_S(\phi _S^S(x_3)(2x_3-3)-\,\phi _S^T(x_3)(2x_3-1)]\nonumber \\&\times E_{af}(t_e)h_{af}((1-x_3),x_2(1-r_D^2),b_2,b_3)\nonumber \\&+\,[\phi _S(x_3)((r_D^2-1)x_2+r_S^2(2x_2-1))\nonumber \\&+,2r_Dr_S(x_2+1)\phi _S^S(x_3)]\nonumber \\&\cdot E_{af}(t_f)h_{af}(x_2,(1-x_3)(1-r_D^2),b_3,b_2)\Bigg \}. \end{aligned}$$

For the nonfactorizable diagrams (g) and (h), the corresponding amplitudes are given as follows:

22 $$\begin{aligned} \mathcal {M}_\mathrm{anf}= & {} -16\sqrt{\frac{2}{3}}\pi C_f m_B^4\int _0^1\mathrm{d}x_1\mathrm{d}x_2\mathrm{d}x_3\nonumber \\&\times \int _0^{1/\Lambda }b_1\mathrm{d}b_1b_2\mathrm{d}b_2 \phi _B(x_1,b_1)\phi _D(x_3,b_2)\nonumber \\&\times \Bigg \{[\phi _S(x_3)(r_D^2+r_S^2(2x_2+x_3-1)-x_2)\nonumber \\&+\,r_Dr_S(\phi _S^S(x_3)(x_2-x_3+3)\nonumber \\&+\,\phi _S^T(x_3)(1-x_2-x_3))]E_\mathrm{anf}(t_g)h_g(x_i,b_1,b_2)\nonumber \\&+\,[\phi _S(x_3)(r_D^2(x_2+2x_3-2)-x_3+1)\nonumber \\&-\,r_Dr_S(\phi _S^S(x_3)(x_2-x_3+1)\nonumber \\&+\,\phi _S^T(x_3)(x_2+x_3-1))]E_\mathrm{anf}(t_h)h_h(x_i,b_1,b_2).\nonumber \\ \end{aligned}$$

For these diagrams with a scalar meson emitted in Fig. 2, the decay amplitudes are expressed as

23 $$\begin{aligned} \mathcal {M}_\mathrm{ef}^{\prime }= & {} 8\pi C_f f_S m_B^4 \int _0^1 \mathrm{d}x_1\mathrm{d}x_3\nonumber \\&\times \int _0^{1/\Lambda }b_1db_1b_3db_3\phi _B(x_1,b_1)\phi _D(x_3,b_3) \nonumber \\&\times \Bigg \{[r_D(2x_3-1)-(1+x_3)]E_\mathrm{ef}(t_a)h_\mathrm{ef}(x_1,x_3,b_1,b_3)\nonumber \\&-\,r_DE_\mathrm{ef}(t_b)h_ef(x_3,x_1,b_3,b_1)\Bigg \}, \end{aligned}$$

24 $$\begin{aligned} \mathcal {M}_\mathrm{enf}^{\prime }= & {} -16\sqrt{\frac{2}{3}}\pi C_f m_B^4\int _0^1\mathrm{d}x_1\mathrm{d}x_2\mathrm{d}x_3\nonumber \\&\times \int _0^{1/\Lambda }b_1\mathrm{d}b_1b_2\mathrm{d}b_2\phi _B(x_1,b_1)\phi _D(x_3,b_1)\phi _S(x_2)\nonumber \\&\times \Bigg \{[r_D^2(2x_2+x_3-2)-r_Dx_3-x_2+1]\nonumber \\&\times E_\mathrm{enf}(t_c^{\prime })h_{enf1}^{\prime }(x_i,b_i)\nonumber \\&+\,[x_2(2r_D^2+r_S^2-1)+x_3(r_D^2+r_D\nonumber \\&+\,2r_S^2-1)]E_\mathrm{enf}(t_d^{\prime })h_\mathrm{enf2}^{\prime }(x_i,b_i)\Bigg \}. \end{aligned}$$

From Eq. (23), one finds that the amplitudes of factorizable emission diagrams with a scalar meson emitted are proportional to the vector decay constant of the scalar, so it will be highly suppressed by the tiny vector decay constant of the scalar meson, or even vanishes for the decays emitting a neutral scalar meson, because the neutral scalar meson cannot be produced through $(V-A)$ current.

For the $B\rightarrow \overline{D}^*S$ decays, there are only the longitudinal polarization contributions in the light of the conservation of angular momentum. The expressions of the factorizable emission contributions can be obtained by the following substitutions in Eq. (19):

25 $$\begin{aligned}&\mathcal {M}_\mathrm{ef}\rightarrow -\mathcal {M}^L_\mathrm{ef},\quad \phi _D\,\rightarrow \,\phi ^{L} _{D^*},\nonumber \\&\quad m_D\,\rightarrow \,m_{D^*},\quad f_D\rightarrow f_{D^*}^L. \end{aligned}$$

For the hard-scattering emission diagrams, the decay amplitudes can be expressed as

26 $$\begin{aligned} \mathcal {M}_\mathrm{enf}^L= & {} 16\sqrt{\frac{2}{3}}\pi C_f m_B^4\int _0^1\mathrm{d}x_1\mathrm{d}x_2\mathrm{d}x_3\nonumber \\&\times \int _0^{1/\Lambda }b_1\mathrm{d}b_1b_2\mathrm{d}b_2\phi _B(x_1,b_1)\phi _{D^*}^L(x_2,b_2)\nonumber \\&\times \Big \{\Big [\phi _S(x_3)(r_D^2(1-2x_2)+r_S^2(x_2+x_3-1)\nonumber \\&+\,\left( \frac{1}{2}r_S^2-1\right) (x_2-1))\nonumber \\&-r_Sx_3(\phi _S^S(x_3)-\phi _S^T(x_3))\Big ]E_\mathrm{enf}(t_c)h_{enf1}(x_i,b_i)\nonumber \\&+\,\Big [\phi _S(x_3)\left( x_2\left( r_D^2+\frac{3}{2}r_S^2-1\right) \right. \nonumber \\&\left. +x_3\left( 2r_D^2+\frac{1}{2}r_S^2-1\right) \right) \nonumber \\&+\,r_Sx_3(\phi _S^S(x_3)+\phi _S^T(x_3))\Big ]E_\mathrm{enf}(t_d)h_\mathrm{enf2}(x_i,b_i)\Big \}.\nonumber \\ \end{aligned}$$

Similarly, the annihilation type contributions can be written as

27 $$\begin{aligned} \mathcal {M}_{af}^L= & {} 8\pi C_f f_B m_B^4\int _0^1\mathrm{d}x_2\mathrm{d}x_3\nonumber \\&\times \int _0^{1/\Lambda }b_2\mathrm{d}b_2b_3\mathrm{d}b_3\phi ^L_{D^*}(x_2,b_2)\nonumber \\&\times \Big \{\Big [\phi _S(x_3)(r_D^2(1-2x_3)+\left( 1-\frac{1}{2}r_S^2\right) (x_3-1))\nonumber \\&+r_Dr_S(\phi _S^S(x_3)+\phi _S^T(x_3))\Big ] h_{af}((1-x_3),x_2(1\nonumber \\&-\,r_D^2),b_2,b_3)E_{af}(t_e)\nonumber \\&+\,\Big [\phi _S(x_3)((1-rd_D^2)x_2+r_S^2\left( 1-\frac{3}{2}x_2\right) )\nonumber \\&+\,2r_Dr_S(1-x_2)\phi _S^S(x_3)\Big ]h_{af}(x_2,(1-x_3)(1\nonumber \\&-\,r_D^2),b_3,b_2)E_{af}(t_f)\Big \}, \end{aligned}$$

28 $$\begin{aligned} \mathcal {M}_\mathrm{anf}^L= & {} -16\sqrt{\frac{2}{3}}\pi C_f m_B^4\int _0^1\mathrm{d}x_1\mathrm{d}x_2\mathrm{d}x_3\nonumber \\&\times \int _0^{1/\Lambda }b_1\mathrm{d}b_1b_2\mathrm{d}b_2\phi _B(x_1,b_1)\phi _{D^*}^L(x_2,b_2)\nonumber \\&\times \Big \{\Big [\phi _S(x_3)(r_D^2(1-2x_2)-r_S^2(x_2+x_3-1)\nonumber \\&+\,x_2\left( 1-\frac{1}{2}r_S^2\right) )\nonumber \\&-\,r_Dr_S((x_2+x_3-1)\phi _S^S(x_3)+(1-x_2\nonumber \\&+\,x_3)\phi _S^T(x_3))\Big ]h_g(x_{i},b_1,b_2)E_\mathrm{anf}(t_g)\nonumber \\&+\,\Big [\phi _S(x_3)(r_D^2(x_2-2x_3+2)+\left( 1+\frac{1}{2}r_S^2\right) (x_3-1))\nonumber \\&-\,r_Dr_S((x_2+x_3-1)\phi _S^S(x_3)+(x_2-x_3\nonumber \\&+\,1)\phi _S^T(x_3))\Big ]h_h(x_{i},b_1,b_2)E_h(t_h)\Big \}. \end{aligned}$$

For those diagrams with a scalar meson emitted in $B\rightarrow \overline{D}^* S$ decays, the factorizable emission contributions can be obtained from Eq. (23) directly by adopting the same substitutions as Eq. (25), and the hard-scattering emission contributions can be expressed as

29 $$\begin{aligned} \mathcal {M}_\mathrm{enf}^{\prime L}= & {} 16\sqrt{\frac{2}{3}}\pi C_f m_B^4\int _0^1\mathrm{d}x_1\mathrm{d}x_2\mathrm{d}x_3\nonumber \\&\times \int _0^{1/\Lambda }b_1\mathrm{d}b_1b_2\mathrm{d}b_2\phi _{B}(x_1,b_1)\phi _{D^*}^L(x_3,b_1)\phi _S(x_2)\nonumber \\&\times \Big \{\Big [r_D^2(x_2-x_3-2)+r_Dx_3+r_S^2x_2\nonumber \\&+\,(1-x_2)\left( 1+\frac{1}{2}r_S^2\right) \Big ]E_\mathrm{enf}(t_c^{\prime })h_{enf1}^{\prime }(x_i,b_i)\nonumber \\&+\,\Big [x_2\left( r_D^2-1-\frac{1}{2}r_S^2\right) +x_3(r_D^2+r_D\nonumber \\&+\,\frac{3}{2}r_S^2-1)\Big ]E_\mathrm{enf}(t_d^{\prime })h_\mathrm{enf2}^{\prime }(x_i,b_i)\Big \}. \end{aligned}$$

As for the Wilson coefficients of each decay modes, they are the same as those of $B_q \rightarrow \overline{D}^{(*)}_{(s)} T$ decays, and can be found in Ref. [63].

## Numerical results and discussions

In this section, we will present the numerical results and some phenomenological analyses of the considered $B\rightarrow \overline{D}^{(*)} S$ decays. First of all, we should list the input parameters in our numerical calculations [1]:

30 $$\begin{aligned} \Lambda _{\overline{MS}}^{f=4}= & {} 0.25\pm 0.05 \mathrm {GeV},\quad m_{B_{(s)}}=5.28(5.37)\mathrm {GeV},\nonumber \\ m_b= & {} 4.8\mathrm {GeV},\nonumber \\ m_{D_{(s)}}= & {} 1.87/1.97\mathrm {GeV},\quad m_{D_{(s)}^*}=2.00/2.11\mathrm {GeV},\nonumber \\ \tau _{B^{\pm /0}}= & {} 1.64/1.52 ps,\quad \tau _{B_{s}}=1.48 ps,\nonumber \\ V_{cb}= & {} 0.0412_{-0.0005}^{+0.0011},\quad V_{us}=0.22534\pm 0.00065,\nonumber \\ V_{ud}= & {} 0.97427\pm 0.00015 . \end{aligned}$$

For the decay constants of the scalar mesons calculated within the QCD sum rules, we adopt the values in Ref. [6].

**Table 1:** Branching fractions of $B_{q} \rightarrow \overline{D}S(a_0(980),\kappa , \sigma , f_0(980))$ decays calculated in the PQCD approach in S1

Decay modes	Class	BRs($10^{-6}$)
$B^{+}\rightarrow \overline{D}^{0}a_{0}^+$	C	$483_{-215-68-12}^{+244+64+26}$
$B^{0}\rightarrow D^{-}a_0^+$	T	$17.6_{-7.6-7.7-0.4}^{+9.8+11.4+0.9}$
$B^{0}\rightarrow \overline{D}^{0}a_{0}^0$	C	$160_{-75-31-4}^{+88+35+9}$
$B^{0}\rightarrow \overline{D}^{0}\sigma (f_n)$	C	$134_{-55-39-3}^{+65+32+7}$
$B^{0}\rightarrow \overline{D}^{0}f_0 (f_n)$	C	$78.4_{-36.2-36.8-1.9}^{+42.8+33.6+4.3}$
$B^{0}\rightarrow D_s^{-}\kappa ^{+}$	E	$72.6_{-22.1-8.8-1.8}^{+24.3+8.0+4.0}$
$B_{s}\rightarrow \overline{D}^{0}\overline{\kappa }$	C	$262_{-131-66-7}^{+154+57+14}$
$B_s\rightarrow D_s^- a_0^+$	T	$64.3_{-28.7-10.2-1.6}^{+42.3+11.0+3.5}$
$B^{+}\rightarrow \overline{D}^{0} \kappa ^{+}$	C	$10.8_{-6.4-1.9-0.3}^{+7.9+1.9+0.7}$
$B^{0}\rightarrow D^{-}\kappa ^+$	T	$4.83_{-1.64-0.63-0.14}^{+1.92+0.73+0.29}$
$B^{0}\rightarrow \overline{D}^{0}\kappa$	C	$6.89_{-4.28-2.51-0.21}^{+5.26+2.36+0.41}$
$B_{s}\rightarrow D^{-}a_{0}^{+}$	E	$3.42_{-1.17-0.44-0.10}^{+1.31+0.41+0.21}$
$B_{s}\rightarrow \overline{D}^{0}a_{0}$	E	$1.70_{-0.58-0.21-0.05}^{+0.65+0.20+0.10}$
$B_{s}\rightarrow \overline{D}^{0}\sigma (f_n)$	E	$1.13_{-0.40-0.14-0.04}^{+0.42+0.17+0.07}$
$B_{s}\rightarrow \overline{D}^{0}\sigma (f_s)$	C	$14.2_{-6.7-2.7-0.4}^{+7.9+2.5+0.9}$
$B_{s}\rightarrow \overline{D}^{0}f_0 (f_n)$	E	$1.36_{-0.45-0.15-0.04}^{+0.51+0.15+0.08}$
$B_s \rightarrow \overline{D}^0 f_0 (f_s)$	C	$10.6_{-5.3-2.6-0.3}^{+6.1-2.5-0.6}$
$B_{s}\rightarrow D_{s}^{-}\kappa ^{+}$	T	$1.02_{-0.47-0.29-0.03}^{+0.59+0.29+0.06}$

**Table 2:** Branching fractions of $B_{(s)}\rightarrow \overline{D} S (a_0(1450), K_0^*(1430), f_0(1370)$, and $f_0(1500))$ calculated in the PQCD approach in S1 and S2, respectively

Decay modes	Class	BRs($10^{-5}$)$S_1$	BRs($10^{-5}$)$S_2$
$B^{+}\rightarrow \overline{D}^{0}a_{0}^+(1450)$	C	$72.1_{-31.6-12.5-1.8}^{+39.2+13.9+3.9}$	$123_{-64-14-4}^{+73+12+5}$
$B^{0}\rightarrow D^{-}a_0^+(1450)$	T,E	$4.09_{-2.09-0.86-0.10}^{+2.63+1.59+0.23}$	$0.92_{-0.43-0.24-0.02}^{+0.57+0.41+0.05}$
$B^{0}\rightarrow \overline{D}^{0}a_0(1450)$	C	$31.3_{-14.3-5.6-0.8}^{+17.4+4.8+1.7}$	$66.2_{-32.8-7.1-1.7}^{+37.6+6.4+3.6}$
$B^{0}\rightarrow \overline{D}^{0}f_0(1370) (f_n)$	C	$28.6_{-12.0-3.4-0.8}^{+13.7+2.5+1.5}$	$16.3_{-7.6-4.0-0.4}^{+9.1+4.9+0.8}$
$B^{0}\rightarrow \overline{D}^{0}f_{0}(1500) (f_n)$	C	$27.2_{-11.1-2.8-0.7}^{+13.2+2.6+1.5}$	$13.2_{-5.9-2.6-0.3}^{+7.4+3.9+0.7}$
$B^{0}\rightarrow D_s^{-}K_{0}^{*+}(1430)$	E	$1.41_{-0.46-0.46-0.03}^{+0.53+0.46+0.08}$	$9.08_{-3.76-0.58-0.22}^{+4.21+0.63+0.50}$
$B_{s}\rightarrow \overline{D}^{0}\overline{K}_0^{*}(1430)$	C	$53.9_{-23.0-6.2-1.3}^{+26.8+3.8+2.9}$	$68.8_{-35.4-8.5-1.7}^{+38.3+7.6+3.8}$
$B_{s}\rightarrow D_s^{-}a_0^{+}$(1450)	T	$10.3_{-4.9-1.8-0.3}^{+5.8+1.9+0.5}$	$4.35_{-2.43-0.97-0.11}^{+3.28+1.21+0.23}$
$B^{+}\rightarrow \overline{D}^0 K_0^{*+}(1430)$	C	$4.72_{-1.83-0.94-0.15}^{+2.22+1.03+0.28}$	$4.96_{-3.15-0.39-0.15}^{+4.02+0.98+0.29}$
$B^{0}\rightarrow {D}^- K_0^{*+}(1430)$	T	$0.97_{-0.36-0.12-0.03}^{+0.45+0.14+0.06}$	$0.79_{-0.38-0.11-0.03}^{+0.46+0.14+0.05}$
$B^{0}\rightarrow \overline{D}^0 K_0^{*0}(1430)$	C	$3.39_{-1.27-0.46-0.10}^{+1.47+0.43+0.21}$	$3.19_{-2.34-0.40-0.09}^{+3.21+0.44+0.20}$
$B_{s}\rightarrow {D}^- a_0^{+} (1450)$	E	$0.14_{-0.07-0.01-0.01}^{+0.06+0.01+0.01}$	$0.43_{-0.18-0.03-0.01}^{+0.21+0.02+0.02}$
$B_{s}\rightarrow \overline{D}^0 a_0^{0}(1450)$	E	$0.07_{-0.03-0.01-0.01}^{+0.03+0.01+0.01}$	$0.21_{-0.09-0.01-0.01}^{+0.11+0.02+0.02}$
$B_{s}\rightarrow \overline{D}^0 f_0(1370)(f_n)$	E	$0.05_{-0.02-0.01-0.01}^{+0.04+0.01+0.01}$	$0.17_{-0.07-0.01-0.01}^{+0.09+0.01+0.01}$
$B_{s}\rightarrow \overline{D}^0 f_0(1370)(f_s)$	C	$2.30_{-1.12-0.25-0.07}^{+1.46+0.15+0.14}$	$2.97_{-1.53-0.32-0.09}^{+2.99+0.30+0.18}$
$B_{s}\rightarrow \overline{D}^0 f_0(1500)(f_n)$	E	$0.05_{-0.02-0.01-0.01}^{+0.04+0.01+0.01}$	$0.17_{-0.07-0.01-0.01}^{+0.10+0.01+0.01}$
$B_{s}\rightarrow \overline{D}^0 f_0(1500)(f_s)$	C	$2.24_{-1.08-0.23-0.07}^{+1.39+0.18+0.13}$	$2.71_{-2.04-0.22-0.08}^{+2.81+0.26+0.17}$
$B_{s}\rightarrow D_s^- K_0^{*+}(1430)$	T,E	$0.36_{-0.18-0.08-0.01}^{+0.24+0.13+0.02}$	$0.39_{-0.16-0.04-0.01}^{+0.21+0.04+0.02}$

**Table 3:** Branching fractions of $B_{q} \rightarrow \overline{D}^* S(a_0,\kappa , \sigma , f_0)$ decays calculated in the PQCD approach in S1

Decay modes	Class	BRs($10^{-6}$)
$B^{+}\rightarrow \overline{D}^{*0}a_{0}^+$	C	$520_{-188-160-13}^{+215+127+29}$
$B^{0}\rightarrow D^{*-}a_0^+$	T	$250_{-84-41-7}^{+91+63+13}$
$B^{0}\rightarrow \overline{D}^{*0}a_{0}$	C	$128_{-67-40-3}^{+76+34+7}$
$B^{0}\rightarrow \overline{D}^{*0}\sigma (f_n)$	C	$171_{-70-54-4}^{+78+45+9}$
$B^{0}\rightarrow \overline{D}^{*0}f_0 (f_n)$	C	$119_{-51-50-4}^{+57+48+6}$
$B^{0}\rightarrow D_s^{*-}\kappa ^{+}$	E	$13.0_{-4.3-2.5-0.4}^{+4.5+1.8+0.7}$
$B_{s}\rightarrow \overline{D}^{*0}\overline{\kappa }$	C	$320_{-153-80-8}^{+178+69+17}$
$B_s\rightarrow D_s^{*-} a_0^+$	T	$169_{-63-52-4}^{+73+56+10}$
$B^{+}\rightarrow \overline{D}^{*0} \kappa ^{+}$	C	$8.80_{-4.41-3.67-0.27}^{+5.78+3.09+0.53}$
$B^{0}\rightarrow D^{*-}\kappa ^+$	T	$3.42_{-1.32-0.98-0.10}^{+1.58+1.44+0.21}$
$B^{0}\rightarrow \overline{D}^{*0}\kappa$	C	$9.25_{-5.03-3.55-0.27}^{+6.54+3.03+0.56}$
$B_{s}\rightarrow D^{*-}a_{0}^{+}$	E	$0.76_{-0.28-0.15-0.03}^{+0.30+0.14+0.04}$
$B_{s}\rightarrow \overline{D}^{*0}a_{0}$	E	$0.38_{-0.15-0.08-0.02}^{+0.14+0.06+0.02}$
$B_{s}\rightarrow \overline{D}^{*0}\sigma (f_n)$	E	$0.19_{-0.08-0.04-0.01}^{+0.07+0.03+0.01}$
$B_{s}\rightarrow \overline{D}^{*0}\sigma (f_s)$	C	$16.4_{-7.6-3.4-0.5}^{+8.8+2.9+1.0}$
$B_{s}\rightarrow \overline{D}^{*0}f_0 (f_n)$	E	$0.27_{-0.10-0.06-0.01}^{+0.10+0.04+0.01}$
$B_s \rightarrow \overline{D}^{*0} f_0 (f_s)$	C	$12.7_{-6.0-3.4-0.3}^{+7.1+3.1+0.8}$
$B_{s}\rightarrow D_{s}^{*-}\kappa ^{+}$	T	$6.83_{-2.92-1.80-0.20}^{+3.43+2.08+0.41}$

In Tables 1, 2, 3 and 4, we tabulate our numerical results of the branching fractions with uncertainties. Considering the four-quark picture beyond the conventional quark model, we cannot calculate the decay constants and the distribution amplitudes within the QCD sum rules and the lattice QCD approach. So, for the light scalars below 1 GeV, we only list the results of S1, i.e., the two-quark scenario. For the heavy scalars, the numerical results in the two scenarios are both presented. Indeed, previous studies [5, 6] indicated that S2 is favorable. Of course, there are many uncertainties in our calculations, and here we mainly evaluate three kinds. The first errors are caused by nonperturbative parameters, such as the decay constants $f_B$, $f_S$, $\overline{f}_S$, the shape parameters $\omega _{B/D}$ in distribution amplitudes of *B* / *D* mesons, the Gegenbauer moments $B_i$ in the distribution amplitudes of the scalar mesons. The second ones are from the unknown next-to-leading order (NLO) corrections, characterized by the choice of the $\Lambda _\mathrm{QCD}(0.25\pm 0.05\mathrm{GeV})$ and the variations of the factorization scales *t* ($0.75t\rightarrow 1.25t$). NLO contributions in PQCD are not available. In Refs. [100, 101], the authors have estimated the NLO effects in $B\rightarrow \pi \pi$ decays by including the NLO form factors, NLO Wilson coefficients, vertex corrections, the quark loops, and the chromomagnetic penguin. The results showed that the NLO effects are modest and the uncertainties due to the scale variation in the LO are reasonable. The last errors come form the uncertainties of the CKM matrix elements listed in Eq. (30). In our calculations, we admit that the next-leading power ($\bar{\Lambda }/m_B$) corrections are not included, which have been estimated to be no more than 10% [55, 56]. Moreover, the next-leading power corrections of $\bar{\Lambda }/m_D$ are even larger. Yet now, all next-leading power corrections are not available, so we will not include them here and leave them as our future work. From the tables, it is apparent that the most significant theoretical uncertainties are from the nonperturbative parameters. In these tables, in order to indicate the dominant contributions, we also mark each channel by the symbols “T” (color-allowed tree contributions), “C” (color-suppressed tree contributions), and “E” (*W* exchange type contributions). Because all of these decays only occur through tree operators, the *CP* asymmetries of these decays are null in SM.

From the tables, one can find that, compared with the $\Delta S =0$ processes, the $\Delta S =1$ processes are all suppressed by the CKM matrix elements $|V_{us}/V_{ud}|^2$. For these T-type decays with a scalar meson emitted, the contributions from factorizable emission diagrams are either suppressed by the tiny vector decay constant of the scalar meson or even vanish for the neutral scalar mesons, though they have large Wilson coefficients. For the two hard-scattering diagrams ((c) and (d) in Fig. 2), because the light-cone distribution amplitude $\phi _S$ of the scalar meson is antisymmetric, the contributions of these two diagrams no longer cancel but are enhanced, which can be seen from Eqs. (24) and (29). So, although the hard-scattering diagrams are suppressed by the Wilson coefficient $C_1$, they also provide sizable contributions and even dominate the decay amplitudes in some decay modes. In addition, we note that, for the T-type decays with $\kappa /K_0^{*}(1430)$ emission, the contributions from factorizable emission diagrams are still sizable, because the vector decay constants of $\kappa /K_0^{*}(1430)$ are not too small due to the mass difference of the up and strange quarks.

**Table 4:** Branching fractions of $B_{(s)}\rightarrow \overline{D}^* S (a_0(1450), K_0^*(1430), f_0(1370)$, and $f_0(1500))$ calculated in the PQCD approach in S1 and S2, respectively

Decay modes	Class	BRs($10^{-5}$)($S_1$)	BRs($10^{-5}$)($S_2$)
$B^{+}\rightarrow \overline{D}^{*0}a_{0}^+(1450)$	C	$207_{-81-23-5}^{+90+24+11}$	$98.2_{-56.1-29.2-2.5}^{+70.1+28.9+5.3}$
$B^{0}\rightarrow D^{*-}a_0^+(1450)$	T	$26.8_{-11.1-7.3-0.6}^{+12.7+7.5+1.5}$	$11.3_{-5.9-2.7-0.2}^{+7.4+2.1+0.7}$
$B^{0}\rightarrow \overline{D}^{*0}a_0(1450)$	C	$40.1_{-17.3-5.3-1.0}^{+21.2+5.2+2.1}$	$58.9_{-31.2-8.4-1.4}^{+36.7+7.7+3.3}$
$B^{0}\rightarrow \overline{D}^{*0}f_0(1370) (f_n)$	C	$44.8_{-18.4-4.0-1.1}^{+21.3+2.1+2.4}$	$27.8_{-21.4-6.0-0.7}^{+32.0+6.7+1.6}$
$B^{0}\rightarrow \overline{D}^{*0}f_{0}(1500) (f_n)$	C	$44.5_{-18.1-3.4-1.0}^{+21.2+2.6+2.5}$	$25.1_{-20.1-4.6-0.6}^{+30.0+5.7+1.4}$
$B^{0}\rightarrow D_s^{*-}K_{0}^{*+}(1430)$	E	$0.53_{-0.17-0.17-0.01}^{+0.22+0.19+0.03}$	$0.99_{-0.49-0.21-0.03}^{+0.56+0.28+0.05}$
$B_{s}\rightarrow \overline{D}^{*0}\overline{K}_0^{*}(1430)$	C	$73.0_{-30.7-5.9-1.8}^{+36.5+4.0+4.0}$	$79.0_{-55.6-11.6-2.0}^{+72.0+9.6+4.3}$
$B_{s}\rightarrow D_s^{*-}a_0^{+}$(1450)	T	$23.0_{-10.1-7.8-0.6}^{+11.1+8.9+1.2}$	$6.20_{-3.62-1.64-0.16}^{+4.58+1.46+0.33}$
$B^{+}\rightarrow \overline{D}^{*0} K_0^{*+}(1430)$	C	$10.1_{-3.3-1.1-0.3}^{+3.9+1.3+0.6}$	$3.08_{-2.25-0.71-0.08}^{+3.62+0.82+0.18}$
$B^{0}\rightarrow {D}^{*-} K_0^{*+}(1430)$	T	$0.75_{-0.24-0.27-0.02}^{+0.26+0.32+0.05}$	$0.09_{-0.07-0.02-0.01}^{+0.14+0.02+0.01}$
$B^{0}\rightarrow \overline{D}^{*0} K_0^{*0}(1430)$	C	$4.90_{-1.81-0.51-0.14}^{+2.08+0.44+0.30}$	$3.80_{-2.79-0.60-0.11}^{+3.80+0.61+0.23}$
$B_{s}\rightarrow {D}^{*-} a_0^{+} (1450)$	E	$0.06_{-0.03-0.02-0.01}^{+0.03+0.01+0.01}$	$0.05_{-0.02-0.01-0.01}^{+0.03+0.01+0.01}$
$B_{s}\rightarrow \overline{D}^{*0} a_0^{0}(1450)$	E	$0.03_{-0.01-0.01-0.01}^{+0.01+0.01+0.01}$	$0.03_{-0.01-0.01-0.01}^{+0.01+0.01+0.01}$
$B_{s}\rightarrow \overline{D}^{*0} f_0(1370)(f_n)$	E	$0.02_{-0.01-0.01-0.01}^{+0.01+0.01+0.01}$	$0.02_{-0.01-0.01-0.01}^{+0.01+0.01+0.01}$
$B_{s}\rightarrow \overline{D}^{*0} f_0(1370)(f_s)$	C	$3.29_{-1.53-0.21-0.10}^{+1.94+0.18+0.20}$	$3.43_{-2.56-0.45-0.11}^{+3.50+0.39+0.20}$
$B_{s}\rightarrow \overline{D}^{*0} f_0(1500)(f_n)$	E	$0.02_{-0.01-0.01-0.01}^{+0.01+0.01+0.01}$	$0.02_{-0.01-0.01-0.01}^{+0.01+0.01+0.01}$
$B_{s}\rightarrow \overline{D}^{*0} f_0(1500)(f_s)$	C	$3.25_{-1.49-0.19-0.09}^{+1.90+0.23+0.20}$	$3.21_{-2.43-0.36-0.10}^{+3.32+0.34+0.19}$
$B_{s}\rightarrow D_s^{*-} K_0^{*+}(1430)$	T	$1.40_{-0.50-0.41-0.04}^{+0.55-0.44-0.09}$	$0.24_{-0.20-0.08-0.01}^{+0.27+0.06+0.01}$

We now discuss the C-type decays with a $\overline{D}^{(*)}$ meson emitted. The factorizable emission diagrams are suppressed by the small Wilson coefficients $C_1+C_2/3$. Since the cancellation between the hard-scattering emission diagrams (*c* and *d* in Fig. 1) is suppressed by the mass difference between the $\overline{c}$ quark and the “light” quark in the emitted $\overline{D}^{(*)}$ meson, the contributions of the hard-scattering emission diagrams with the large Wilson coefficient $C_2$ are no longer negligible; even they dominate the decay amplitudes. Therefore, their branching fractions are expected to be large enough to be detected at on-going experiments, especially for these $\Delta S =0$ processes.

As is well known, the annihilation type diagrams are power suppressed in the PQCD approach, and the branching fractions of the E-type decays are expected to be much smaller than the others. However, for the $B \rightarrow \overline{D}^{(*)}S$ decays, because there is a large mass difference between the $\overline{D}$ meson and the scalar meson, which will weaken the cancellation between the two nonfactorizable annihilation type diagrams (g) and (h) in Fig. 1, the contributions of the annihilation diagrams might be sizable. As a result, the branching fractions of these E-type decays are not too small, as usual, especially for these $\Delta S =0$ processes. For example, enhanced by the CKM matrix elements, the branching fractions of the $B^0 \rightarrow D_s^- K_0^{*+}(800/1430)$ even reach $10^{-5}$, the order of which is measurable in the on-going experiments. When the experimental data is available, it will provide another platform to study the dynamical mechanism of the annihilation diagrams in two-body hadronic *B* decays.

Specially, $B^{+} \rightarrow \overline{D}^{(*)0}a_0^+(980)$ and $B^+\rightarrow \overline{D}^{(*)0}\kappa ^+(800)$ decays have both a T-type contribution with a scalar meson emitted and a C-type contribution with $\overline{D}^{(*)}$ emitted. For $B^+\rightarrow \overline{D}^{(*)0} a_0^+(980)$ decays, the constructive interference between those two contributions makes the branching fractions larger than the pure C-type decays, such as $B_s \rightarrow \overline{D}^{(*)0} \overline{\kappa }^0$ decay. Similarly, the constructive (destructive) interferences also lead the branching fractions of $B^+\rightarrow \overline{D}^{(*)0}\kappa ^+$ larger (smaller) than the pure C-type $B^0 \rightarrow \overline{D}^{(*)0} \kappa ^0$ decays. In particular, because the vector decay constant of $\kappa$ is not tiny, the T-type contributions with a $\kappa$ emitted are sizable. From the Table 1, one can also find that $\mathcal {B} (B_s \rightarrow D_s^- a_0^+ (980))>\mathcal {B}(B^0\rightarrow D^- a_0(980)^+)$ and $\mathcal {B}(B^0\rightarrow D^- \kappa ^+(800))>\mathcal {B}(B_s \rightarrow D_s^- \kappa ^+(800))$, which can be understood by the interference between T-type and E-type contributions. The relations $\mathcal {B}(B^0\rightarrow D^{*-} a_0(980)^+) > \mathcal {B} (B_s \rightarrow D_s^{*-} a_0^+ (980))$ and $\mathcal {B}(B_s \rightarrow D_s^{*-} \kappa ^+(800))>\mathcal {B}(B^0\rightarrow D^{*-} \kappa ^+(800))$ in Table 3 can also be explained in the same manner.

From Tables 2 and 4, it is found that, for these C-type decays, such as $B_s \rightarrow \overline{D}^{(*)0} \overline{K}_0^{*0}(1430)$ and $B^0 \rightarrow \overline{D}^{(*)0} K_0^{*0}(1430)$ decays, the branching fractions in S1 are roughly equal to those in S2. This can be explained by the fact that the two dominant nonfactorizable diagrams (c) and (d) in Fig. 1 will be cancelled out by each other. Thus, the effects caused by the wave functions of scalar mesons are inconspicuous. In fact, such cases also occur in the color-suppressed $B\rightarrow D^{(*)} S$ decays in Ref. [50]. We also note that the branching fraction of $B^+\rightarrow \overline{D}^0 a_0^+(1450)$ in S2 is larger than that in S1. That is because the constructive interference between the C-type contributions with $\overline{D}$ emitted and the T-type contributions with the scalar meson emitted in S2 is much larger than that in S1. However, for the decays $B^+\rightarrow \overline{D}^{*0} a_0^+(1450)$ and $B^+ \rightarrow \overline{D}^{*0} K_0^{*+}(1430)$, the destructive interference causes their branching fractions in S2 to be smaller than those in S1. As for $B^+ \rightarrow \overline{D}^0 K_0^{*+}(1430)$, although the interference between the two type contributions is also constructive in S2, its branching fraction in S2 is only slightly larger than that in S1, because the contributions from factorizable diagrams and the nonfactorizable ones are canceled out by each other, especially when the vector decay constant of $K_0^{*+}(1430)$ is no longer as small as the others. We also note that the $B^+\rightarrow \overline{D}^{(*)0} a_0^+(1450)$ decays can be used to identify different scenarios. Similarly, for the decays $B^0\rightarrow \overline{D}^{(*)0} a_0^0(1450)$ and $B^0\rightarrow \overline{D}^{(*)0} f_0(1370/1500)$, the branching fraction differences between the two scenarios are ascribed to the interference between the emission contributions and the annihilation ones.

The T-type $B_s \rightarrow D_s^{(*)-} a_0^+(1450)$ decays, which are pure emission processes with $a_0^+(1450)$ emitted, are dominated by the hard-scattering emission diagrams, since the factorizable diagrams are highly suppressed by the small vector decay constant of the $a_0^+(1450)$. The ratio of branching fractions between S1 and S2 is about 2 and 4, for $B_s \rightarrow D_s^{-} a_0^+(1450)$ and $B_s \rightarrow D_s^{*-} a_0^+(1450)$, respectively, which indicates that the branching fractions are sensitive to the scenarios. From Eq. (20), it is found that the contributions from the two hard-scattering diagrams are enhanced by each other. When we switch from S1 to S2, the changes induced by the distribution amplitudes in S2 will overlap with each other, which makes the branching fractions different from those in S1. As for $B^0 \rightarrow D^{(*)-} a_0^+(1450)$ decays, the ratio between S1 and S2 is about 4 for $B^0 \rightarrow D^{-} a_0^+(1450)$ and about 2 for $B^0 \rightarrow D^{*-} a_0^+(1450)$, which is contrary to the cases in $B_s \rightarrow D_s^{(*)-} a_0^+(1450)$ decays. This is caused by the interferences between the emission diagrams and the annihilation ones. In S2, the interference is destructive for $B^0 \rightarrow D^{-} a_0^+(1450)$ decays, but constructive for $B^0 \rightarrow D^{*-} a_0^+(1450)$. Unlike the cases of the above T-type decays with the $a_0(1450)$, the branching fractions of the $B^0 \rightarrow D^-K_0^{*+}(1430)$ and $B_s \rightarrow D_s^-K_0^{*+}(1430)$ decays in the two scenarios are roughly equal. However, for $B^0 \rightarrow D^{*-}K_0^{*+}(1430)$ and $B_s \rightarrow D_s^{*-}K_0^{*+}(1430)$ decays, the branching fractions in S1 are much larger (about seven–eight times larger) than those in S2. For the above four decays, the color-allowed factorizable emission contributions are sizable in S2, because the vector decay constant of the $K_0^{*+}(1430)$ in S2 is larger than in S1. For $B^0 \rightarrow D^-K_0^{*+}(1430)$ and $B_s \rightarrow D_s^-K_0^{*+}(1430)$ decays, the interference between the above contributions and the ones of the hard-scattering emission diagrams is constructive, so their branching fractions in S2 are roughly equal to those in S1. However, this kind of interference is destructive for $B^0 \rightarrow D^{*-}K_0^{*+}(1430)$ and $B_s \rightarrow D_s^{*-}K_0^{*+}(1430)$ decays, and their branching fractions in S2 are smaller than those in S1.

Now, we turn to a discussion of the pure annihilation decays, which are dominated by the nonfactorizable annihilation diagrams. From Table 2, one can find that the branching fractions of pure E-type $B \rightarrow \overline{D} S$ decays in S2 are much larger than those in S1. As is well known, the cancellation between two nonfactorizable annihilation diagrams is suppressed by the large mass difference between the *b* quark and the light quark. So, the changes induced by the distribution amplitudes of the scalars become important, which leads to the fact that the branching fractions are obviously dependent on the scenarios. Taking $B_s \rightarrow D^- a_0^+(1450)$ for illustration, the branching ratios in S2 are about three times larger than that in S1. However, from Table 4, we find that the situation is reversed for the pure annihilation $B \rightarrow \overline{D}^* S$ decays, the discrepancies of branching fractions in different scenarios are quit small. Comparing the Eq. (21) with Eq. (27), we notice that the two factorizable annihilation diagrams are cancelled by each other in $B \rightarrow \overline{D} S$ decays but enhanced in $B \rightarrow \overline{D}^* S$ decays. So, in $B \rightarrow \overline{D}^* S$ decays, the contributions from two factorizable diagrams are comparable with those from nonfactorizable ones. Moreover, the interference between the factorizale annihilation diagrams and the nonfactorizable ones is destructive (or constructive) in S1 (S2), which causes that the branching fractions in S2 are almost equal to or even larger than those in S1.

In the conventional two-quark picture for the light scalars, although the LHCb experiment had measured the branching fractions of $B(B_s) \rightarrow \overline{D} \sigma$ and $\overline{D}f_0(980)$ [48, 49], the mixing angle $\theta$ cannot be constrained stringently due to the large uncertainties. For convenience, we present individually the branching fractions under the pure $n\bar{n}$ and $s\bar{s}$ components in the tables. Once the S1 is confirmed and the mixing angle is fixed, one can obtain the branching fractions directly from the two predictions with the $n\bar{n}$ and $s\bar{s}$ components. For instance, if the popular value ranges $[25^{\circ }, 40^{\circ }]$ and $[140^{\circ }, 165^{\circ }]$ are adopted, we can predict the branching fractions as listed in Table 5. In the same manner, by neglecting the tiny glueball contents and adopting the results of Eq. (11), we also list the branching fractions of the decay modes with $f_0(1370)$ or $f_0(1500)$ in Table 6. Note that we here only list the center values for simplicity.

**Table 5:** The calculated branching fractions of $B_{(s)}\rightarrow \overline{D}^{(*)} f_0(980)$ and $\sigma$ with the mixing in the PQCD approach (unit:$10^{-6}$)

Decay modes	$[25^{\circ }, 40^{\circ }]$	$[140^{\circ }, 165^{\circ }]$
$B^{0}\rightarrow \overline{D}^{0}\sigma$	$78.6\sim 110$	$78.6\sim 125$
$B^{0}\rightarrow \overline{D}^{0}f_0(980)$	$46.0\sim 64.4$	$46.0\sim 73.1$
$B_s\rightarrow \overline{D}^{0}\sigma$	$3.99\sim 7.22$	$1.66\sim 5.87$
$B_s\rightarrow \overline{D}^{0}f_0(980)$	$5.46\sim 7.92$	$8.09\sim 10.6$
$B^{0}\rightarrow \overline{D}^{*0}\sigma$	$100\sim 140$	$100\sim 159$
$B^{0}\rightarrow \overline{D}^{*0}f_0(980)$	$70.2\sim 98.2$	$70.2\sim 111$
$B_{s}\rightarrow \overline{D}^{*0}\sigma$	$2.93\sim 6.70$	$1.37\sim 7.09$
$B_{s}\rightarrow \overline{D}^{*0}f_0(980)$	$7.62\sim 10.5$	$7.55\sim 11.9$

**Table 6:** The calculated branching fractions of $B_{(s)}\rightarrow \overline{D}^{(*)} f_0(1370)$ and $f_0(1500)$ with the mixing in the PQCD approach (unit:$10^{-6}$)

Decay modes	S1	S2
$B^{0}\rightarrow \overline{D}^{0}f_0(1370)$	173	99.2
$B^{0}\rightarrow \overline{D}^{0}f_0(1500)$	79.3	38.5
$B_s\rightarrow \overline{D}^{0}f_0(1370)$	7.40	4.85
$B_s\rightarrow \overline{D}^{0}f_0(1500)$	14.9	24.5
$B^{0}\rightarrow \overline{D}^{*0}f_0(1370)$	272	168
$B^{0}\rightarrow \overline{D}^{*0}f_0(1500)$	129	73.2
$B_{s}\rightarrow \overline{D}^{*0}f_0(1370)$	10.7	7.55
$B_{s}\rightarrow \overline{D}^{*0}f_0(1500)$	20.8	24.6

In fact, under the two-quark assumption, only the $n\bar{n}$ component contributes to the decay modes $B^0 \rightarrow \overline{D}^0 f_0(980)$ and $B^0\rightarrow \overline{D}^0 \sigma$. Thus, we can define the ratio

31 $$\begin{aligned} r=\frac{B^0 \rightarrow \overline{D}^0 f_0(980)}{B^0\rightarrow \bar{D}^0 \sigma }=\frac{\sin ^2 \theta }{\cos ^2 \theta }=\tan ^2\theta . \end{aligned}$$

Using the latest experimental data in Eq. (1), we can obtain $r=0.12^{+0.09}_{-0.06}$, which can constrain the range of the mixing angle as

32 $$\begin{aligned} \theta \in [14^{\circ },24^{\circ }] \quad \text {or}\quad [155^{\circ },166^{\circ }]. \end{aligned}$$

Compared with the results of Ref. [80–85], the obtuse angle solutions agree with each other, but the acute angle we obtained is a bit smaller than the previous results. Using the mixing angle value in Eq. (32) and the results in Table 1, we get the branching fractions of $B^0 \rightarrow \overline{D} f_0(80)/\sigma$:

33 $$\begin{aligned} \mathcal {B}(B^0\rightarrow \overline{D}^0 \sigma )\sim & {} (11.9_{-0.8}^{+0.7}) \times 10^{-5},\nonumber \\ \mathcal {B}(B^0\rightarrow \overline{D}^0 f_0(980)\sim & {} (0.8_{-0.4}^{+0.5}) \times 10^{-5}, \end{aligned}$$

where the errors are only from the mixing angle. Compared to Eq. (1), one can find that our numerical results can accommodate the experimental data well within the limit of errors.

It should be noted that there are large uncertainties in our results and discussions, especially ones induced by the nonperturbative parameters, which might lower our prediction power and blur the distinction between the two scenarios even in favorable cases. Therefore more reliable nonperturbative approaches and experimental data are needed for describing the nature of the scalar mesons in the future.

## Summary

Motivated by recent results of the charmed *B* decays with a scalar from LHCb experiment, we attempted to investigate the $B/B_s \rightarrow \overline{D}^{(*)} S$ decays induced by the $\overline{b} \rightarrow \overline{c}$ transition within the framework of the PQCD approach. Although the light scalar mesons, especially $f_0(980)$ and $a_0 (980)$, are widely perceived as primarily four-quark bound states, in practice it is difficult to make quantitative predictions based on the four-quark picture for light scalars. Hence, predictions are made in the two-quark model for the decays with the light scalar mesons. For the decays with scalar mesons above 1 GeV, we have explored two possible scenarios, the difference depending on whether the light scalars are treated as the lowest lying $q\bar{q}$ states or four-quark particles. By comparing with the experimental data, we can deduce whether the heavy scalars are the ground states or the first excited ones, though the current studies prefer the former. Since the considered decays occur only through tree operators, there are no *CP* asymmetries. The branching fractions of most decay modes are in the range of $10^{-4}$–$10^{-7}$, which can be tested in the LHCb experiment and by Belle-II in the near future. Some decays have large branching fractions, such as the $B^+ \rightarrow \overline{D}^{(*)0} a_0^+(980/1450)$ and $B^+ \rightarrow D^{(*)-} a_0^+(980/1450)$, which are measurable in the current experiment. For the $B^0 \rightarrow \overline{D}^0 \sigma$ and $B^0 \rightarrow \overline{D}^0 f_0(980)$ decays, our numerical results accommodate the experimental data well within the limit of the errors, even in the two-quark picture. We note that although the nonperturbative parameters, the higher-order and high-power correction, and even the effects of the final states interaction lead rather large uncertainties, the orders of magnitude and some phenomenological discussions will be useful for the on-going LHCb and the forthcoming Belle-II experiments.
